# Knowledge domain and emerging trends in multimorbidity and frailty research from 2003 to 2023: a scientometric study using citespace and VOSviewer

**DOI:** 10.1186/s13561-023-00460-9

**Published:** 2023-10-10

**Authors:** Penghong Deng, Chang Liu, Mingsheng Chen, Lei Si

**Affiliations:** 1 School of Health Policy & Management, Nanjing Medical University, Jiangning District, Nanjing, 211166 China; 2 Center for Global Health, Nanjing Medical University, Nanjing, China; 3 School of Health Sciences, Western Sydney University, Campbelltown, Australia; 4 Translational Health Research Institute, Western Sydney University, Penrith, Australia

**Keywords:** Multimorbidity, Frailty, Scientometric, Bibliometric, CiteSpace, VOSviewer

## Abstract

**Background:**

Multimorbidity and frailty represent emerging global health burdens that have garnered increased attention from researchers over the past two decades. We conducted a scientometric analysis of the scientific literature on the coexistence of multimorbidity and frailty to assess major research domains, trends, and inform future lines of research.

**Methods:**

We systematically retrieved scientific publications on multimorbidity and frailty from the Web of Science Core Collection, spanning from 2003 to 2023. Scientometric analysis was performed using CiteSpace and VOSviewer, enabling the visualization and evaluation of networks comprising co-citation references, co-occurring keywords, countries, institutions, authors, and journals.

**Results:**

A total of 584 eligible publications were included in the analysis. An exponential rise in research interest in multimorbidity and frailty was observed, with an average annual growth rate of 47.92% in publications between 2003 and 2022. Three major research trends were identified: standardized definition and measurement of multimorbidity and frailty, comprehensive geriatric assessment utilizing multimorbidity and frailty instruments for older adults, and the multifaceted associations between these two conditions. The United States of America, Johns Hopkins University, Fried LP, and the Journal of the American Geriatrics Society were identified as the most influential entities within this field, representing the leading country, institution, author, and journal, respectively.

**Conclusions:**

Scientometric analysis provides invaluable insights to clinicians and researchers involved in multimorbidity and frailty research by identifying intellectual bases and research trends. While the instruments and assessments of multimorbidity and frailty with scientific validity and reliability are of undeniable importance, further investigations are also warranted to unravel the underlying biological mechanisms of interactions between multimorbidity and frailty, explore the mental health aspects among older individuals with multimorbidity and frailty, and refine strategies to reduce prescriptions in this specific population.

**Supplementary Information:**

The online version contains supplementary material available at 10.1186/s13561-023-00460-9.

## Background

Population ageing is rapidly intensifying worldwide, from 461 million people older than 65 years in 2004 to an estimated 2 billion by 2050 [1], leading to profound implications for the planning and delivery of health policy and social care. An ageing population makes it possible to accumulate multiple chronic diseases, a condition termed multimorbidity, commonly defined as two or more chronic conditions coexisting within the same individual [2, 3]. Its estimated prevalence among the middle-aged and elderly ranges from 30 to 82%, depending on the definition used and the population investigated [4]. Likewise, as an important geriatric term, frailty is recognized as an accumulation of biological deficiencies, characterized by an increased susceptibility to stressors due to the declined reserves and functions of multiorgan systems [5]. A multinational epidemiological survey reported the prevalence of frailty ranging between 12% and 24% among community-dwelling older people [6]. However, investigations of multimorbidity and frailty have been conducted predominantly in high-income countries, and their prevalence is possibly underestimated due to the absence of surveillance capacity in low- and middle-income countries (LMICs) for comprehensive counts of potential cases [6, 7].

Despite the fact that multimorbidity and frailty represent two distinct concepts, both are complex syndromes characteristics of aging. The occurrence and development of multimorbidity and frailty are closely correlated because of a certain degree of biological overlap, and the two conditions may coexist or occur successively within the same elderly [8, 9]. Compared to the general elderly population, individuals with multimorbidity are more likely to experience frailty in latter life, and similarly, frail patients are often at a high risk of multiple comorbidities [9, 10]. Given the potential bidirectional causality between the two conditions, the current study is focused on a wide range of research on the coexistence of multimorbidity and frailty and their relationship. In addition, both multimorbidity and frailty are important risk factors for mortality in older adults, which has been demonstrated in numerous studies and across various settings and subpopulation [11, 12]. The two conditions are also associated with a broad range of other adverse outcomes, including disability [13], falls [14], fractures [15], depression [16], lower quality of life [17], cognitive impairment [18], dementia [19], and hospitalization [20]. Additionally, the long-term and continuous care required for multimorbidity and frailty and their associated complications contribute to the increase in emergency, outpatient and inpatient costs [21, 22]. As life expectancy continues to increase globally, multimorbidity and frailty are without question among the most serious global health problems, and continue to impose a massive health and economic burden on individuals, families, healthcare systems, and society.

In response to the emerging scientific challenges, multimorbidity and frailty have garnered significant research interest and attention over the past two decades. Initially developed and employed in a public health context, the concept of multimorbidity focuses on the structure of coexisting chronic conditions [2]. On the other hand, frailty represents a geriatric notion that requires a comprehensive evaluation of both the individual and their environment [8]. The development and implementation of diagnostic and screening instruments for multimorbidity and frailty have greatly enhanced their application in primary health care and geriatric settings [8, 11]. Guidelines for the care of individuals with multimorbidity and frailty have been further developed and refined, considering the intersection of physical and mental health disorders, the aging process, and polypharmacy [23]. Furthermore, the terms multimorbidity and frailty often appear simultaneously and are increasingly used in medical literature as indicators of health and risk profiles among older adults, providing support for clinical decision-making and the design of targeted interventions [11, 13, 17, 18, 24]. Significant progress has been made in the management and promotion of multimorbid and frail health. While existing publications have provided insights into specific aspects of multimorbidity and frailty, however, a synthesis of the knowledge and priorities of the research on multimorbidity and frailty over decades has not yet been conducted.

Considering the increase in publications over recent decades, a comprehensive scientometric analysis has the potential to offer a snapshot of the research domain and valuable insights into the research priorities within this field, particularly in the settings of the ageing population and high prevalence of multimorbidity and frailty. Complementary to systematic reviews and meta-analyses, scientometric analysis is a sophisticated application of bibliometrics that enables broadly synthesized quantitative analysis of scientific research, answering the fundamental question of “what is studied” [25, 26]. Importantly, this approach can reveal prevailing trends within a given field and generate predictions for future research with integrated scientific concepts and methodological tools [27, 28]. It is particularly pertinent for further progress in aging research, and for research groups desiring collaborations and focusing on the latest research trends. Furthermore, the integration of visualization and data mining techniques has enhanced the scientometric approach, which has not yet been extensively used in scientometric studies on multimorbidity and frailty. Therefore, the primary objective of this study was to conduct a scientometric analysis to gauge the intellectual evolution, trends, and future prospects of research on the coexistence of multimorbidity and frailty and their association over the past 20 years. Our secondary objectives included evaluating research networks among countries, institutions, authors, and journals, as well as measuring collaborations, research performance, and identifying gaps within the field.

## Methods

### Data source

The literature used in this study was retrieved from the Web of Science Core Collection (WOSCC). WOSCC is a comprehensive collection of high-quality academic journals and literature from around the world [29]. It offers powerful indexing functions that go beyond basic information such as authors, affiliations, journals, countries or regions, and keywords [29]. It also includes a comprehensive citation network and information, making it a preferred and highly reliable source for scientometric studies [29]. For this study, we specifically extracted publications associated with multimorbidity and frailty from the Science Citation Index Expanded (SCIE) and the Social Science Citation Index (SSCI).

### Retrieval strategy and data collection

**Fig. 1:**
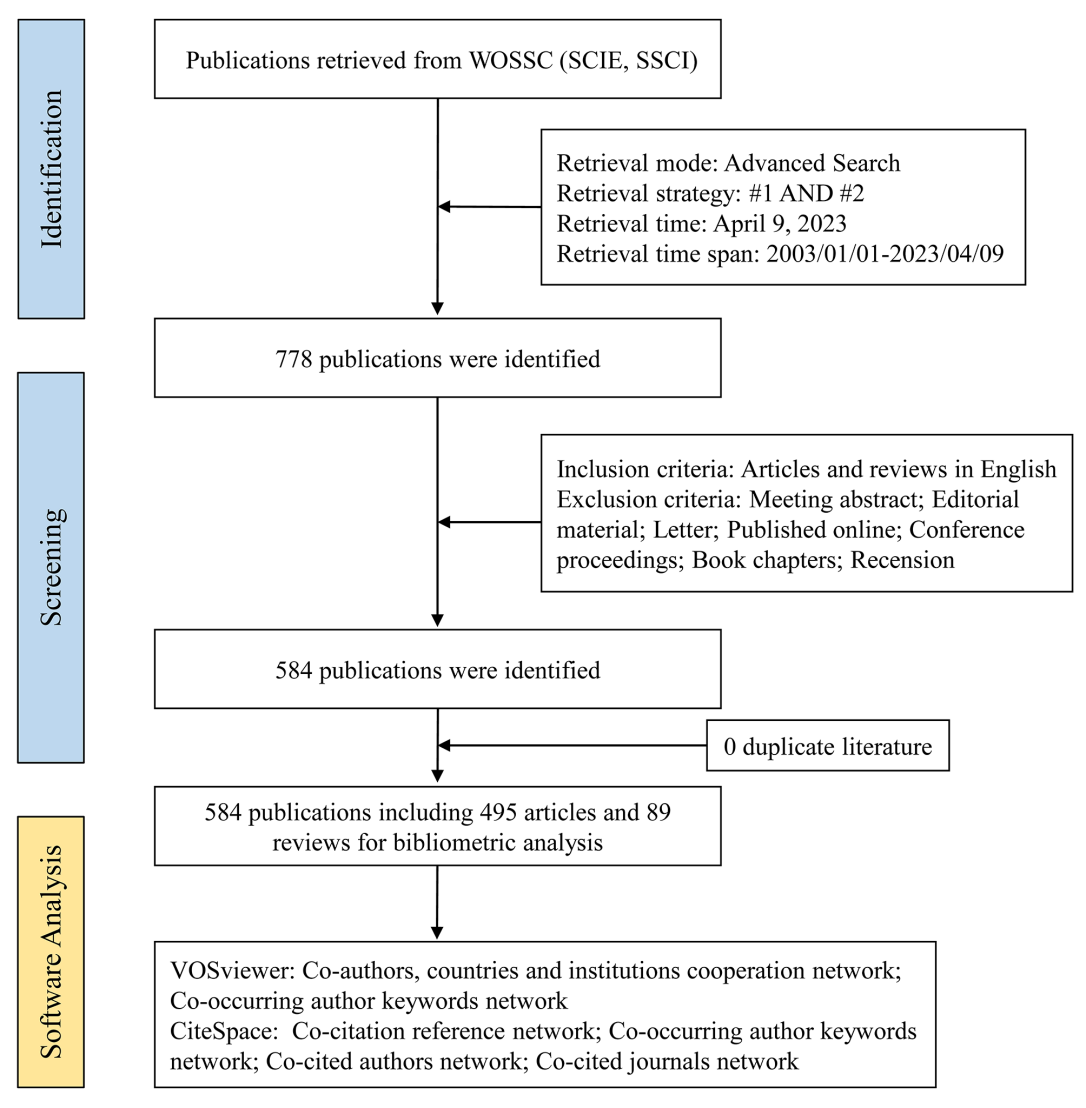
Flow chart of the scientometric study. #1: (TI= (multimorbidit* or multi-morbidit* or ‘multiple morbidities’ or multiple-morbidities or comorbidit* or co-morbidit* or ‘multi* disease*’ or ‘multi* chronic disease*’ or ‘multi* chronic condition*’) OR AK= (multimorbidit* or multi-morbidit* or ‘multiple morbidities’ or multiple-morbidities or comorbidit* or co-morbidit* or ‘multi* disease*’ or ‘multi* chronic disease*’ or ‘multi* chronic condition*’)); #2: (TI= (frail* or debilit* or weak*) OR AK= (frail* or debilit* or weak*)). Abbreviations: WOSCC, Web of Science Core Collection

An advanced retrieval was conducted by the same investigator (P.D.) on a single day (April 9, 2023), to minimize bias resulting from daily literature updates. To ensure the high relevance of the literature to the topic, a combination of title (TI) and author keywords (AK) was used for the retrieval. The final search strategy employed was as follows: (TI= (multimorbidit* or multi-morbidit* or ‘multiple morbidities’ or multiple-morbidities or comorbidit* or co-morbidit* or ‘multi* disease*’ or ‘multi* chronic disease*’ or ‘multi* chronic condition*’) OR AK= (multimorbidit* or multi-morbidit* or ‘multiple morbidities’ or multiple-morbidities or comorbidit* or co-morbidit* or ‘multi* disease*’ or ‘multi* chronic disease*’ or ‘multi* chronic condition*’)) AND (TI= (frail* or debilit* or weak*) OR AK= (frail* or debilit* or weak*)). The search covered the period from January 1, 2003, to April 9, 2023, and was limited to publications in English and the publication types of ‘article’ or ‘review’. Exclusion criteria were specified, and the retrieved documents were deduplicated. The process is illustrated in Fig. 1.

### Measures

We employed two techniques to investigate research evolution and trends:

1. Co-citation network of references: The co-citation network is based on the relationship between two documents being cited by a third document at a specific time, representing the intellectual foundation of the third document [30]. As the subject evolves, the co-citation network expands from a single network to multiple networks, illustrating the shifts in the intellectual foundation over time [30]. These transitions reflect the research tracks and trends in the citing documents. By analyzing the co-citation reference network, an intellectual landscape is constructed using highly cited literature and research frontiers (identified by extracting themes from the citing literature).
2. Co-occurring network of author keywords: Keywords provide insights into the specific research areas and directly address the research hotspots within the field [28]. The co-occurrence network measures the frequency of paired keywords within a collection of documents and captures their associations. The process of co-occurrence analysis involves extracting keywords from the documents, tallying keyword frequencies, and identifying clusters, bursts, and connections among keywords [28].

As for our secondary objectives, we constructed collaborative networks of countries, institutions, authors, and journal co-citation networks. These networks help identify high-impact journals, reveal connections, and provide insights into the distribution of disciplinary knowledge domains. Additionally, author co-citation analysis was performed to identify highly cited authors, examine their connections, and explore the corresponding intellectual structure within the field [28, 31].

### Software and data analysis

In our analysis, we utilized two software tools: VOSviewer (version 1.6.19) and CiteSpace (version 6.2.R2 advanced), as described by van Eck & Waltman and Chen et al. [28, 31]. VOSviewer, developed by Waltman et al. (2010), is a program specifically designed for constructing and analyzing networks and generating bibliometric maps in a user-friendly and visually appealing manner [31]. We employed VOSviewer to analyze the networks of authors’ countries, institutions, co-author collaborations, and co-occurring keywords. CiteSpace, on the other hand, is a Java application developed by Chen et al. (2009) that enables the visualization and analysis of scientific documents [28]. Its primary objective is to detect emerging trends within an intellectual field. CiteSpace integrates systematic mapping, bibliometric analysis based on citation analysis theories, data mining algorithms, and scientometrics to investigate a research domain [28]. Bibliometrics is a classic approach of information analysis based on mathematics and statistics that enables researchers to better understand the structure and linkages of evidence [27]. Systematic mapping provides a research snapshot of current knowledge, enabling the identification of areas ready for full synthesis and those requiring more research focus. By utilizing CiteSpace, we were able to identify intellectual bases, hotspots, trends, and bursts within the field.

The time slice for scientific literature analysis in CiteSpace was set to one year. The g-index is an author-level metric based on the distribution of citations that reduces bias from highly cited papers. The scale factor k, the determinant of g-index, was set to the recommended value of 25 to grant credibility to both high and low cited papers. Cluster labels were extracted from keywords lists using the log-likelihood ratio algorithm (*P* < 0.001). The networks generated by VOSviewer and CiteSpace consist of nodes and lines. Nodes represent different entities, such as references, keywords, authors, countries, and institutions, and are clustered into groups based on their similarities. The size of the nodes indicates citation frequencies, occurrences, or centrality, reflecting their importance and impact within the network. The closeness of nodes indicates centrality, and connections between nodes represent collaborations, co-citations, or co-occurrences among them. The colors of both the nodes and links provide information about the year of the corresponding citations, clusters, or occurrences. Highly connected nodes are included between and within clusters, revealing relevant areas and their evolution throughout the years.

Using the structural variant analysis and burst term analysis functions of CiteSpace, we examined important items influencing the structure of mapping networks in the present study, making it possible to assess potential future research directions. In the structural variation analysis, the degree of structural variation introduced by a new article can provide prospective information based on the boundary spanning mechanism [32]. If an article introduces new linkages across different subject boundaries, we expect that it has the potential to bring the knowledge structure to a new turning point, which can be an important bridge and focus for future research [32]. Another key method, burst term detection, is capable of identifying meaningful and bursting structures in the document stream over time based on data streaming algorithms [33]. With the appearance of emerging themes, the frequency and intensity of certain features suddenly increase in recent timespans, which is a signal of promising work ahead [28, 33]. We conducted burst detection analysis on cited references, keywords, authors, and journals to synthesize and reveal possible future research priorities. Additionally, to illustrate the evolutions and connections among clusters, we utilized timeline analysis, which involved distributing nodes within each cluster on a common timeline.

Three key parameters that needed interpretation in relation to the effect of clusters were betweenness centrality, modularity, and silhouette, as outlined by Chen et al. (2010) [28]. Betweenness centrality allowed us to assess the importance of each node by calculating the number of shortest paths between all pairs of nodes, particularly identifying influential nodes within a cluster and pivotal hubs between clusters. The modularity score (Q) indicated the clustering effect of the network, ranging from 0 to 1, with Q > 0.3 denoting a significant division of network clusters. The silhouette score (S) measured the homogeneity within clusters, ranging from − 1 to 1, and S > 0.7 indicated a high level of resemblance among nodes within each cluster. Furthermore, we employed centrality divergence, a measure of the dispersion of betweenness centrality distributions of nodes, to assess the innovation of citing documents in the structural variation network [32].

## Results

Two different software packages were employed to present a comprehensive overview of the advancement of research on multimorbidity and frailty over the past 20 years, comprising the analysis of publication outputs, and the knowledge mapping of co-cited references, author keywords, countries, institutions, authors, and journals.

### Analysis of publication outputs and trends

For the final analysis, a total of 584 unique documents were included, consisting of 495 articles and 89 reviews. These documents received a total of 19,585 citations after the screening and exclusion process. The analysis involved 3,713 co-authors, with an average of 6.36 authors per literature. These authors were affiliated with 2,672 institutions located in 222 countries/territories. The number of publications in this field exhibited a significant increase over time. In 2003, only 2 publications were identified, but this number grew at an average annual growth rate of 47.92%, reaching 87 publications in 2022. However, it should be noted that the annual number of publications and citations showed a declining trend since the analysis was conducted only up until April 2023. While the number of publications per year may appear relatively low, there has been a notable increase in the average number of citations per document. In 2003, the average number of citations per document was 0.5, but by 2023, it had risen to 30 (Figure S1).

### Analysis of co-citation references

#### Clusters of research

**Fig. 2:**
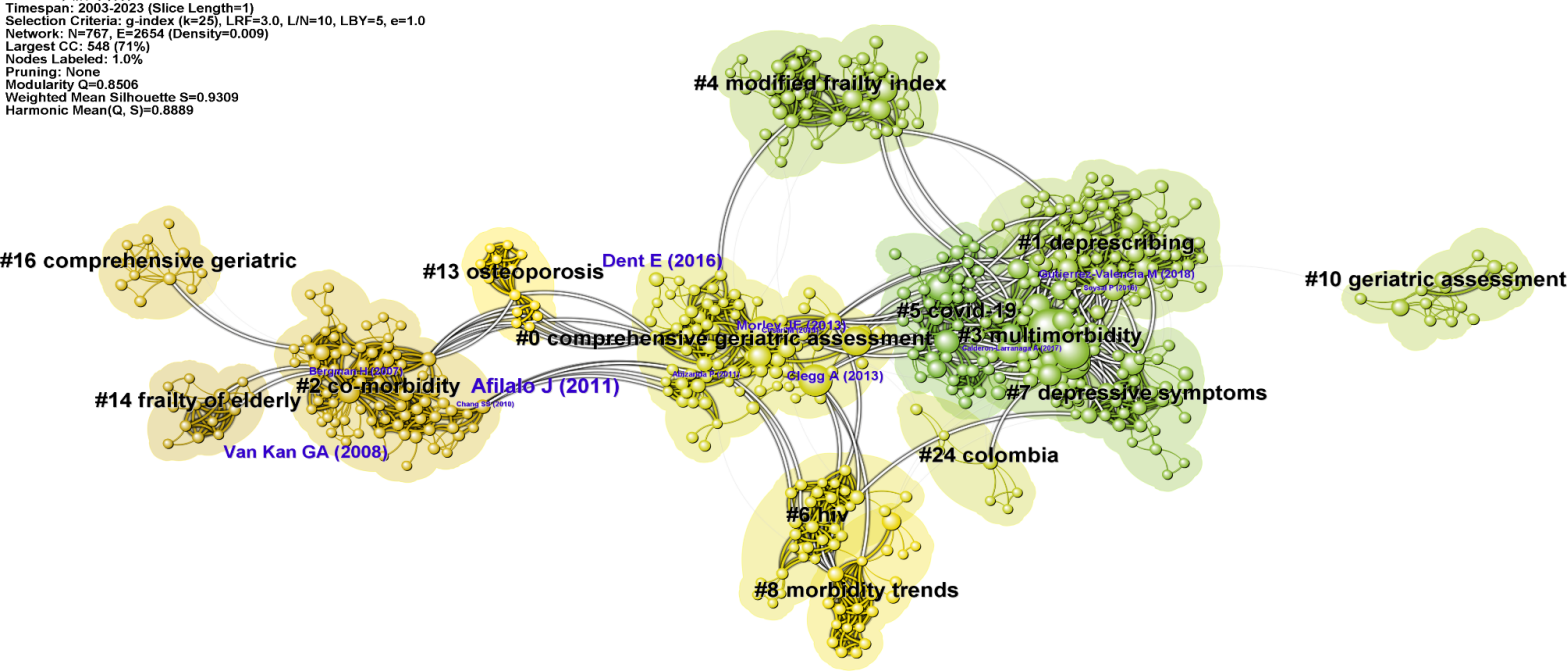
Co-citation references network and corresponding clustering visualization generated by CiteSpace. Note: A node represents a cited reference. The network is organized by the betweenness centrality of every node. The size of a node is proportional to its betweenness centrality. A node with high betweenness centrality is usually one that connects two or more clusters or lies at the core within a cluster, and the correspondent cited literature has a significant impact on the local network. The highlighted lines represent the evolution and connections among different clusters

A total of 24 clusters were identified in the co-citation reference network, demonstrating significant modularity (Q = 0.8506) and high silhouette scores (S = 0.9309), indicating the credibility and distinctiveness of the clusters (Fig. 2). More detailed descriptions of each cluster are available in Table S1. Three major research trends were identified based on the largest linkage pathways between clusters. The clusters contributing to these trends are presented with their cluster label, size, silhouette score, average year of publication, and the most representative reference.

The first major research trend concerned the theoretical conceptualization of multimorbidity and frailty. This trend started in 2006 with cluster #14 (‘frailty of elderly’; 13; S = 0.994; 2006) in our database and a central topical article published by Ahmed et al. in the American Journal of Medicine, which synthesized the resurgence of significance and interest in frailty [5]. This cluster then evolved into cluster #2 (‘co-morbidity’; 76; S = 0.979; 2007) [34], which was considered a physical condition strongly associated with frailty. In this cluster, various definitions and measurements of frailty and comorbidity were proposed and compared, which was an emerging field of ongoing debate [34, 35].

The second major research trend revolved around the comprehensive geriatric assessment (CGA) for older adults using multimorbidity and frailty tools. This trend began with cluster #16 (‘comprehensive geriatric’; 12; S = 0.992; 2007) and presented the third standardized and scientific multidimensional geriatric assessment tool [36]. This cluster then combined with cluster #13 (‘osteoporosis’; 13; S = 0.987; 2012) [37], extending the evaluation tools and applicable population of CGA. Over the past decade, this research field had further enriched and converged into the largest cluster #0 (‘comprehensive geriatric assessment’; 81; S = 0.93; 2013) [38], in line with cluster #10 (‘geriatric assessment’; 16; S = 0.994; 2015) [39], indicating the benefits of CGA as a clinical tool for addressing the medical and functional demands of older adults. Additionally, cluster #6 (‘HIV’; 31; S = 0.954; 2013) [40] and #8 (‘morbidity trends’; 23; S = 0.996; 2010) [41] were dedicated to the assessment of multimorbidity and frailty status in older people living with HIV. Finally, the latest cluster #4 (‘modified frailty index’; 49; S = 0.978; 2015) [42] focused on comorbidity and frailty indexes and their comparison in assessing the risk of adverse outcomes, further contributing to the advancement of CGA tools. Cluster #2 was an essential assembly point between the first trend identified above and the third major trend.

The third research trend focused on the association between multimorbidity and frailty, initially exploring the overlapping definitions among multimorbidity, frailty, and disability. Cluster #2 then evolved into cluster #3 (‘multimorbidity’; 70; S = 0.851; 2017) [9], positioned at the center of the network and forming strong connections with surrounding clusters. This research field delved into the commonalities and interactions between multimorbidity and frailty, particularly their co-adverse effects on the elderly.

In addition, we identified several emerging domains within the analysis. Cluster #1 (‘deprescribing’; 79; S = 0.854; 2017) [43] focused on the benefits of reducing prescribing for critically ill patients. Cluster #5 (‘Covid-19’; 48; S = 0.93; 2019) [44] explored the impacts of multimorbidity and frailty on the elderly with Covid-19. Cluster #7 (‘depressive symptoms’; 30; S = 0.926; 2018) [45] investigated the mental health of multimorbid and frail individuals, including the risk of depression.

**Fig. 3:**
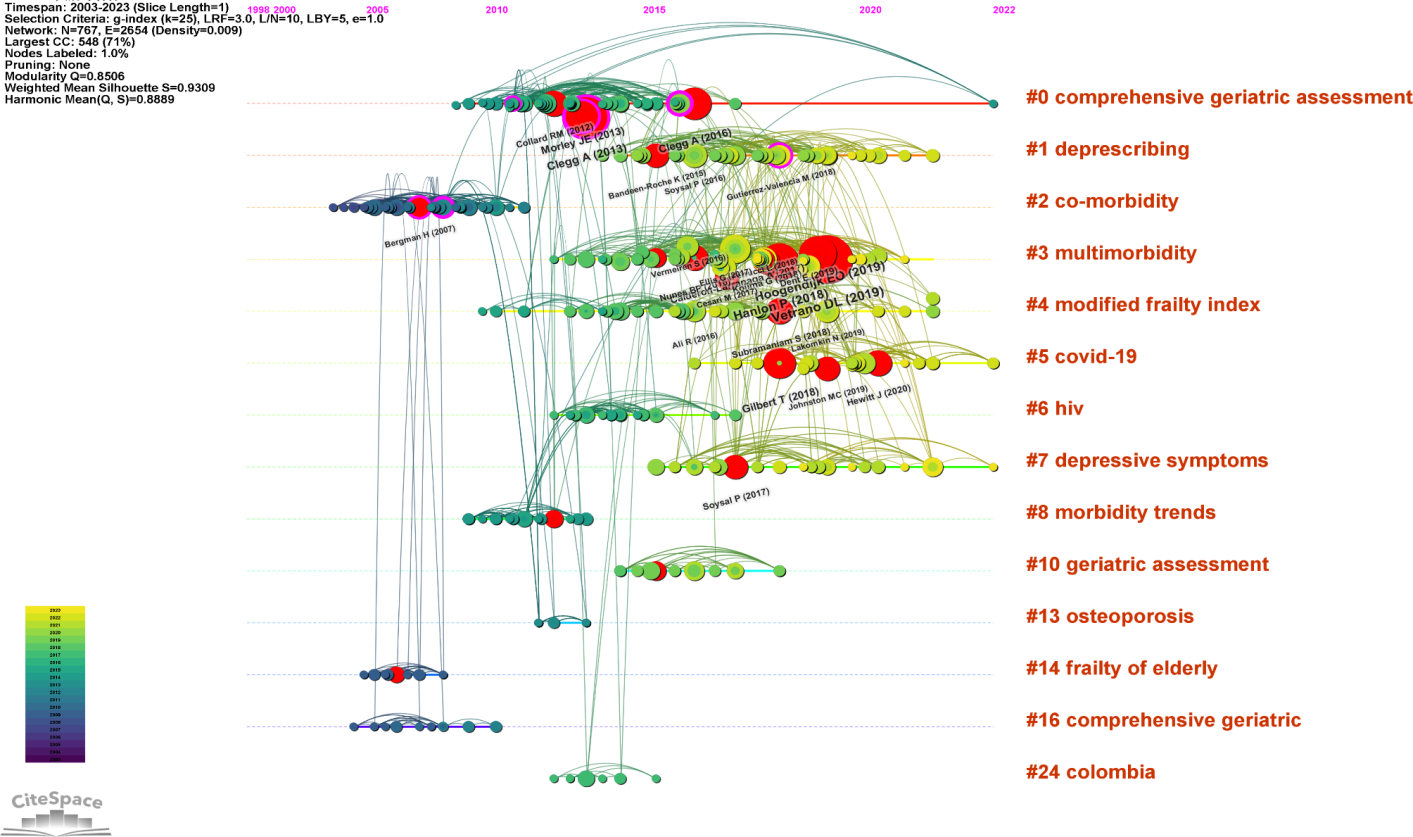
Timeline visualization of co-citation references network. Note: A node represents a cited reference. The size of a node depends on its betweenness centrality. For each cluster, nodes are organized by their year of publication on horizontal lines. Nodes with large coloured tree rings are those with high betweenness centrality (external purple tree rings) and burst strength (central red tree rings). The colour of lines indicate the time of links between nodes or between clusters

The timeline map provided a visual representation of the duration and historical progression of each cluster, effectively capturing the trends mentioned earlier. It also allowed us to pinpoint the temporal placement of landmark publications. Notably, the most recent and dynamically active clusters in the analysis were cluster #1 (‘deprescribing’), cluster #3 (‘multimorbidity’), cluster #4 (‘modified frailty index’), cluster #5 (‘Covid-19’), and cluster #7 (‘depressive symptoms’), indicating a growing research interest in these areas (Fig. 3).

#### Most cited references and transformative papers

Table 1 presents the top ten most cited references, which played a crucial role in shaping the intellectual foundations of the clustering studies. The meta-analysis on frailty and multimorbidity conducted by Vetrano DL et al. in 2019 emerged as the most co-cited paper, with 41 citations within our reference network and a total of 216 citations in the literature [9]. Notably, a comprehensive review on frailty in older adults authored by Clegg et al. in the Lancet received 25 co-citations within our network and an impressive 3364 citations from the literature database [46]. It is worth mentioning that these two publications exhibited substantial bursts of strength, measuring 11.93 and 10.58 respectively, suggesting their potential significant impact on multimorbidity and frailty research (Table S2).

Furthermore, we conducted a structural variation analysis to identify transformative papers that fostered significant advances in the research field through cross-domain connections. The three most transformative citing papers, as indicated by the highest centrality divergence scores, are as follows (Table S3): a new multidimensional scale proposed by Amici A et al. for identifying frailty in the elderly [47], a longitudinal cohort study by Sarkisian et al. to identify sub-dimensions of frailty [48], and a review by Wleklik et al. on the determinants of frailty syndrome [10]. These papers have made significant contributions to the field and have been instrumental in advancing our understanding of multimorbidity and frailty.

**Table 1:** The top 10 most cited references

Number of citations in the network/literature (April 2023)	Year	Title	Source	DOI	Cluster ID
41/216	2019	Frailty and multimorbidity: a systematic review and meta-analysis	J GERONTOL A-BIOL	10.1093/gerona/gly110	#3
28/381	2018	Frailty and pre-frailty in middle-aged and older adults and its association with multimorbidity and mortality: a prospective analysis of 493 737 UK Biobank participants	LANCET PUBLIC HEALTH	10.1016/S2468-2667(18)30091-4	#3
26/838	2019	Frailty: implications for clinical practice and public health	LANCET	10.1016/S0140-6736(19)31786-6	#3
25/3364	2013	Frailty in elderly people	LANCET	10.1016/S0140-6736(12)62167-9	#0
20/689	2016	Development and validation of an electronic frailty index using routine primary care electronic health record data	AGE AGEING	10.1093/ageing/afw039	#0
19/2296	2013	Frailty Consensus: A Call to Action	J AM MED DIR ASSOC	10.1016/j.jamda.2013.03.022	#0
17/532	2018	Development and validation of a Hospital Frailty Risk Score focusing on older people in acute care settings using electronic hospital records: an observational study	LANCET	10.1016/S0140-6736(18)30668-8	#5
15/201	2017	Assessing and Measuring Chronic Multimorbidity in the Older Population: A Proposal for Its Operationalization	J GERONTOL A-BIOL	10.1093/gerona/glw233	#3
12/344	2018	Frailty index as a predictor of mortality: a systematic review and meta-analysis	AGE AGEING	10.1093/ageing/afx162	#3

### Analysis of co-occurring author keywords

**Fig. 4:**
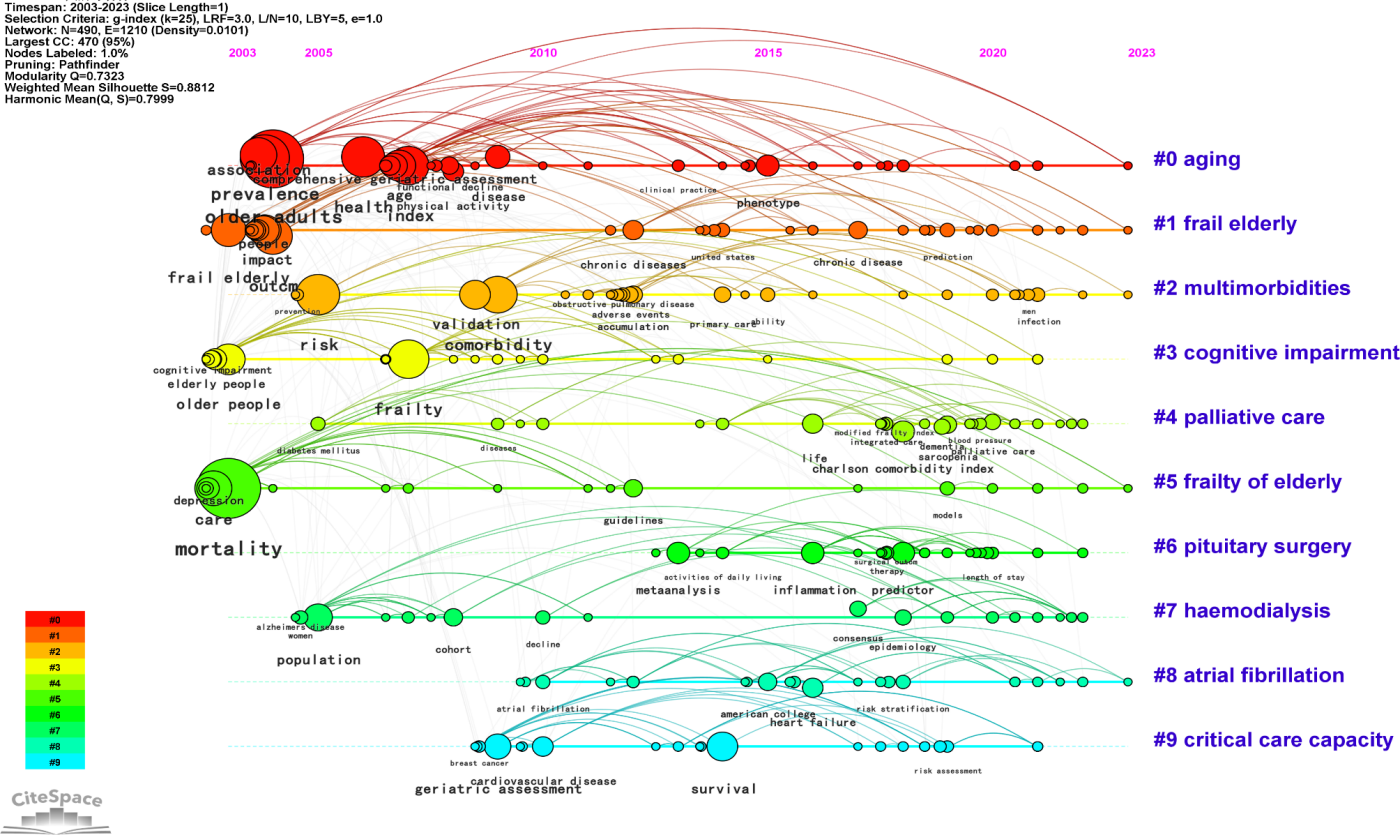
Timeline visualization of co-occurring keywords network. Note: A node represents a keyword. The position of the node corresponds to the year of keyword occurrence. The size of a node is proportional to the frequency of its occurrence. The clusters are labelled in blue on the far right of the timeline map

Figure 4 shows a timeline mapping generated from the co-occurring author’s keyword network using CiteSpace. The keyword clusters and their distributions were deemed plausible, with significant modularity and silhouette scores (S = 0.8812; Q = 0.7323). The analysis extracted the 10 largest keyword clusters, namely cluster #0 (‘aging’; 46; S = 0.9; 2011), #1 (‘frail elderly’; 42; S = 0.861; 2014), #2 (‘multimorbidities’; 37; S = 0.874; 2014), #3 (‘cognitive impairment’; 34; S = 0.885; 2009), #4 (‘palliative care’; 34; S = 0.847; 2018), #5 (‘frailty of elderly’; 33; S = 0.89; 2009), #6 (‘pituitary surgery’; 33; S = 0.787; 2018), #7 (‘haemodialysis’; 32; S = 0.84; 2014), #8 (‘atrial fibrillation’; 28; S = 0.849; 2016), and #9 (‘critical care capacity’; 27; S = 0.881; 2014) (Table S4).

**Fig. 5:**
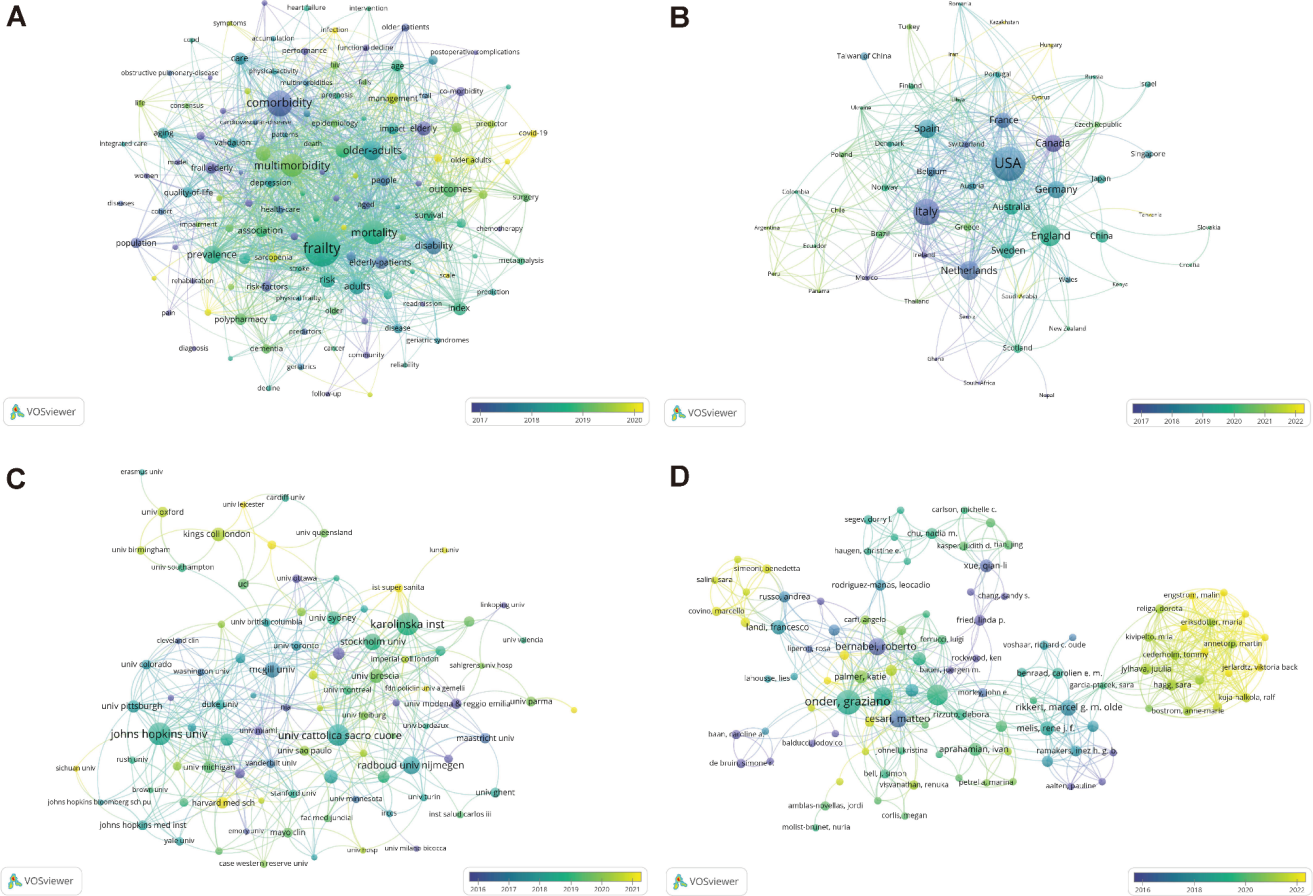
**A**. Network of co-occurring author keywords; **B**. Network of cooperation between countries; **C**. Network of cooperation between institutions; **D**. Network of cooperation between authors. Note: The size of a node is proportional to the frequency of its occurrence. The color of the node corresponds to the average year of publication

Furthermore, a burst analysis was conducted to identify the most dynamic keywords (Table S5). The top three keywords with the strongest burst strength were ‘elderly people’, ‘functional status’, and ‘population’. The top three most durable keywords, ranked by the beginning of citation bursts, were ‘elderly people’, ‘functional status’, and ‘frail elderly’. The keywords ‘infection’ and ‘Charlson comorbidity index’ exhibited the latest bursts, particularly active from 2021 to 2023. To visualize the keyword network, an overlay visualization was performed using VOSviewer, based on the average year of publication. The most cited keywords capturing the research trends were ‘frailty’, ‘comorbidity’, ‘mortality’, and ‘multimorbidity’, aligning with our research theme (Fig. 5A).

### Analysis of collaboration networks across countries and institutions

Figure 5B displays the cooperation networks of countries, while Fig. 5C shows the cooperation networks of institutions. In total, 56 countries or territories were captured in the analysis. The United States of America (USA) held a central position, with the highest number of publications (n = 159), followed by Italy with 96 publications and England with 69 publications. In terms of citations, the USA was also the most cited country (n = 9524), followed by Italy (n = 5004) and Canada (n = 4380) (Table S6). Furthermore, VOSviewer identified 90 institutions from the dataset. Johns Hopkins University emerged as both the most published institution (n = 23) and the most cited institution (n = 5264). Karolinska Institute also produced 23 publications, while the University of Cattolica del Sacro Cuore had 21 publications. In terms of citations, the University of Cattolica del Sacro Cuore ranked second (n = 3327), followed by Dalhousie University (n = 2956) (Table S6).

### Analysis of co-authorship networks

A network of co-cited authors was established, demonstrating significant modularity and silhouette scores (Q = 8490; S = 0.9337) (Figure S2). Cluster #0, titled ‘complex health problems’, emerged as the most significant and central cluster, encompassing research related to multimorbidity, frailty, cognitive impairment, and Covid-19 in older adults. Within this network, Charlson ME, Cesari M, and Covinsky KE were identified as key authors bridging cluster #6 (‘frail elderly’) and cluster #14 (‘multimorbidity’). The top three most cited authors were Fried LP (n = 280), Rockwood K (n = 176), and Charlson ME (n = 119). The top three authors with the strongest betweenness centrality, indicating their influence in connecting different parts of the network, were Onder G (0.20), Inouye SK (0.18), and Mitnitski AB (0.17) (Table S7). Vetrano DL was identified as the top cited author with the strongest burst strength, indicating a significant increase in citations, and the most active author from 2020 to 2023 (Table S8).

Additionally, a collaborative network of citing authors was analyzed, highlighting influential cooperative groups centered around Onder G, Marengoni A, Vetrano DL, Bernabei R, and Cesari M (Fig. 5D). These authors played a key role in the network and contributed significantly to the collaboration and advancement of research in the field.

### Analysis of co-cited journals

We constructed a network of co-cited journals to provide researchers with valuable insights into important knowledge sources and suitable journals for submitting their studies (Figure S3). Among the 585 unique journals identified, the top three most cited journals were the Journal of the American Geriatrics Society (n = 373), Journals of Gerontology Series A-biological Sciences and Medical Sciences (n = 371), and the Lancet (n = 303). These journals have been influential in the field of multimorbidity and frailty research. The top three journals with the highest betweenness centrality, indicating their significance in connecting different parts of the network, were Aging Clinical and Experimental Research (0.33), Annals of Internal Medicine (0.29), and Age and Ageing (0.22) (Table S7). These journals have played a crucial role in the dissemination and exchange of research related to multimorbidity and frailty. Archives of Internal Medicine emerged as the journal with the strongest burst, indicating a significant increase in citations, and it remained active for the longest period, spanning from 2004 to 2017. The latest bursting journals were the Lancet Public Health and World Neurosurgery, suggesting their recent prominence and active engagement in the field (Table S9). Researchers may consider these journals as potential outlets for their research on multimorbidity and frailty.

## Discussion

### Summary of the main findings

Our study provided a comprehensive and insightful snapshot of the knowledge landscape surrounding multimorbidity and frailty, illustrating the connections between evidence and revealing the trends and evolution of research over the past 20 years. Despite a relatively small number of publications per year, there was a discernible growth trend in the literature on multimorbidity and frailty. The co-cited literature network and author’s keyword analysis depicted strong linkages across 24 and 10 different clusters, respectively, collectively highlighting three major research trends: standardized definition and measurement of multimorbidity and frailty, comprehensive geriatric assessment for older adults using multimorbidity and frailty tools, and the multidimensional relationship between the two conditions. Additionally, several emerging trends received increased focus, including the benefits of reducing prescribing for critically ill patients, the mental health of the elderly with multimorbidity and frailty, and the impacts of COVID-19. The United States and Johns Hopkins University emerged as the most prolific country and institution, respectively. Among the authors, Fried LP, Rockwood K, and Charlson ME were the most frequently cited, while Vetrano DL demonstrated recent productivity and activity. The top three most cited journals were the Journal of the American Geriatrics Society, the Journal of Gerontology Series A - Biological Sciences and Medical Sciences, and The Lancet.

### Identification of research trends

The resulting co-citation reference network and author’s keyword analysis extracted three distinct major research trends in multimorbidity and frailty from 2003 to 2023, which were also captured by the qualitative analysis of highly cited literature. The first research trend focused on the theoretical concepts and standardized measurements of multimorbidity and frailty. Previous studies have made significant efforts to standardize the definition of multimorbidity and frailty, including the evolution from the concept of comorbidity to multimorbidity, the definition of the multidimensional domains of frailty, and the pioneering distinction between the concepts of multimorbidity and frailty [2, 5, 8, 44, 49–51]. The diversified definitions and standards have improved the understanding of the elderly health problems and led to an expert consensus for future diagnosis and care, and for research and medical education in this field. Additionally, a range of screening and metric tools for multimorbidity and frailty has been developed based on questionnaires, clinical practice, and routine data, such as the widely used Charlson comorbidity index (CCI), frailty phenotype (FP), and frailty index (FI) [44, 52, 53]. This field is currently progressing towards providing specific instruments for a given environment and population. However, further validation of the clinical validity of these instruments is required.

The second major and influential research trend revolved around CGA, with studies primarily targeting multimorbid and frail individuals. Comorbidity and frailty indexes or scales were used to improve diagnosis and treatment programs, integrate geriatric care, predict adverse outcomes, and increase survival chances among elderly inpatients. CGA represented a multidimensional and multidisciplinary approach to identify the medical, social, and functional conditions of the elderly and develop a series of integrated and coordinated care programs, which contributed to the individual benefits for patients and the sustainability of health care systems [54]. The intricacy and specialized care needs of multimorbidity and frailty made CGA an optimal choice for healthcare. CGA allowed for a broader assessment of problematic areas for multimorbid and frail individuals, including potential polypharmacy, quality of life, and physical and cognitive function, which enabled the development of more specific and individualized care interventions to result in improved overall quality of care [55]. In particular, multimorbidity and frailty were utilized as clinical tools to predict and evaluate the risk profile of the elderly, including hospitalization, complications, cognitive impairment, depression, disability, and mortality, thereby facilitating clinical decision-making and reducing the risk of adverse short-term outcomes [12, 13, 24, 40, 45, 56]. Undoubtedly, the benefits of CGA in geriatric assessment have been well-established.

The third major research trend involved the association between multimorbidity and frailty, encompassing their commonalities, comparisons, and bidirectional effects. In a groundbreaking article published in 2004, Fried and colleagues revealed the underlying relationship between multimorbidity (defined as the presence of two or more chronic conditions in an individual) and frailty (measured by the frailty phenotype), concluding that the two terms were overlapping, coexisting, and interacting [49]. However, it was also identified that multimorbidity or comorbidity could act as a determinant of frailty [10, 57], while frail individuals were more susceptible to developing chronic conditions, leading to a mutually reinforcing cycle [9]. Over the years, extensive research has thoroughly investigated the associations between multimorbidity and frailty. This includes examining the distinctions between the two concepts, exploring the relationship between the number and severity of chronic conditions and the risk of frailty, and investigating the effects of different multimorbidity measurements and patterns on frailty [13, 58–60]. These studies have contributed to a deeper understanding of the complex interplay between multimorbidity and frailty.

### Outputs and influence networks

The analysis of research outputs and influence networks serves as a secondary objective of this study, aiming to capture the distribution, identify gaps, and recognize high-impact countries, research groups, and authors within specific subjects. The provided collaborative and co-cited visualization networks, along with their corresponding performance data, will assist readers, particularly those actively involved in the research, to gain insights into the field. In terms of countries/territories and institutions, the USA and Johns Hopkins University ranked first in both the number of publications and citations, which can be attributed to their top researchers and well-established biomedical foundations. Extensive collaboration was observed between European and American countries and institutions, while developing countries were not prominently represented in terms of influential contributions in this field. It is essential to encourage and support more studies from disadvantaged countries or institutions to gain a better understanding of the interactions of distinct sociocultural factors in multimorbidity and frailty. Furthermore, our co-cited author network highlights the significant contributions of Fried LP to the field, particularly in the definition, diagnosis, and care research of frailty [49]. While the quantitative information obtained from the co-authors’ collaborative network may not fully capture their influence, it is possible to identify scholars who have made significant contributions to multimorbidity and frailty through the analysis of the most cited and transformative literature. It is important to note that the rankings of retrieved journals, based on the number of publications or citations in the WOSCC, do not necessarily reflect the quality of cited papers. However, the analysis of co-cited journals does identify the most cited journals in a given research area, such as the Journal of the American Geriatrics Society in our network, which are considered appropriate for specific topics.

### Potential trends for future research

Despite the identification of multiple research trends, predicting the most influential domains of future research remains challenging due to limitations in bibliometric outputs, such as short-term citation patterns and deferred identifications. However, the current findings on the latest high-cited, transformative, and bursting items provide valuable evidence to support investigations into future trends. Multimorbidity and frailty are closely related but separate constructs that are relevant to a wide range of clinical practitioners and researchers. However, the lack of international standard definitions and measurements with scientific validity and feasibility poses a significant obstacle to translating research evidence into clinical practice [61]. Particularly in the domain of frailty, a consensus on a consistent definition is lacking, especially regarding social and psychological frailty, which are often overlooked [62]. Future research should focus on developing more accurate and accessible tools for clinical caregivers to identify and manage multimorbidity and frailty. The largest cluster of CGA has demonstrated the potential of multimorbidity and frailty measurements in predicting adverse outcomes. However, most comorbidity and frailty models were developed in white populations and might be more applicable to people living in Southern Europe, and in Hispanic and African American older adults [46, 63] Therefore, further validation of their applicability using different cutoffs for multimorbidity and frailty may be necessary in a broader population. It is worth noting that multimorbidity has been identified as a contributor to frailty [51], highlighting the interplay between these two conditions. However, the multidimensional nature of this relationship remains ambiguous due to the lack of clinical trials and longitudinal, evidence-based studies exploring the underlying biomarkers and mechanisms. Future well-designed research is needed to investigate the shared biological pathways contributing to multimorbidity and frailty, examine the longitudinal relationships and mechanisms between multimorbidity patterns and frailty, and explore the effects of multimorbidity on different domains of frailty. Lastly, several emerging reference clusters indicate the need for further attention to reducing inappropriate prescribing for older adults, understanding the impact of multimorbidity and frailty on individuals with Covid-19, and addressing the mental health of older multimorbid patients due to their increased vulnerability to frailty [60]. These areas warrant additional research focus and intervention development.

### Implications for practice and public health

This comprehensive scientometric study of multimorbidity and debility may provide useful information for researchers, grants applicants, funding agencies, and policy decision makers. Multimorbidity and frailty are multifaceted concepts of care needs that involve physical, environmental, and psychological domains [23, 51]. Thus, approaches to the treatment and management of multimorbidity and frailty should be individualized and flexible, taking into account the individual needs, preferences, and health priorities. In the patient-centered care settings, precision care and disease education by a multidisciplinary team are warranted. Given that multimorbidity and frailty are strongly associated with adverse outcomes, instruments for their clinical measurement are of particular importance. However, in population health research, most multimorbidity and frailty instruments have been validated only for outcome prediction, and aspects such as reliability, cross-cultural validity, and responsiveness have received little attention [64, 65]. For the healthcare practitioners, they also need a simple, non-time-consuming assessment of multimorbidity and frailty that supports decision-making on interventions and care distribution. Spanning the entire life course, the relationship between frailty and multimorbidity has been recognized [9]; continuous primary health care interventions and control of risk factors may constrain the adverse progress of frailty and have ancillary benefits for the primary and secondary prevention of multiple diseases. We advocate for a profound transition of research evidence to clinical practice, and multifaceted endeavors can improve the elderly health.

### Strengths and limitations

Compared to a narrative review, scientometric analysis serves as a more comprehensive and systematic approach, offering invaluable insights to clinicians and researchers regarding the research landscape and the emergence of innovative trends. This analytical method enables the identification of scientific inquiries that have remained inadequately explored and addressed, potentially shaping the trajectory of future research endeavors [66]. Moreover, this analysis may help identify the most influential authors, journals, and institutions within the realms of multimorbidity and frailty and promote collaborations and knowledge exchange within specific areas of research. However, several limitations of this study warrant careful consideration. First, co-citation analysis constitutes a crucial component of scientometric analysis; however, citation bias, including publication bias, self-citation, authorship bias, and journal impact factor bias, may potentially compromise the objectivity of the study findings [67]. Second, the data collection was exclusively sourced from the SCIE and SSCI indexed by WOSCC, resulting in a restricted number of retrieved publications. Other mainstream databases such as PubMed and Embase, which provide full-text references and citation lists, were not included [29]. Third, our co-citation network solely extracted the first author, which may inadequately address the influence of other contributing authors. Additionally, the clusters within the co-occurrence network were susceptible to variations in keyword expressions. Last, the co-citation network’s capacity to detect the most recent trends was limited due to the insufficient citation of the latest literature.

## Conclusion

This first scientometric study provides a comprehensive analysis of the historical trends and research landscape pertaining to multimorbidity and frailty. Over the past two decades, the number of publications in this field has steadily increased, with a peak in 2021. The study identifies the most important countries, institutions, authors, and journals in this field, as well as research hotspots and emerging trends, such as standardized concepts and measurements of multimorbidity and frailty, the use of CGA with multimorbidity and frailty instruments, and the multidimensional associations between these conditions. The findings highlight the need for increased cooperation between institutions in Europe, the USA, and China, particularly with influential authors. The study provides valuable information for clinicians and researchers to understand current research trends and future directions in multimorbidity and frailty, and it also informs funding agencies about research priorities related to the coexistence and relationship between multimorbidity and frailty.

### Electronic supplementary material

Below is the link to the electronic supplementary material.

Supplementary Material 1

## Data Availability

The datasets used in this study are available from the corresponding author on reasonable request.

## Abbreviations

LMICs: low- and middle-income countries
WOSCC: Web of Science Core Collection
SCIE: Science Citation Index Expanded
SSCI: Social Science Citation Index
TI: Title
AK: Author keywords
CGA: Comprehensive geriatric assessment
USA: The United States of America
CCI: Charlson comorbidity index
FP: Frailty phenotype
FI: Frailty index
