# Supplementary material for: Knowledge domain and emerging trends in multimorbidity and frailty research from 2003 to 2023: a scientometric study using citespace and VOSviewer
Source: Health Econ Rev. 2023 Oct 10;13:46. doi: 10.1186/s13561-023-00460-9 (PMC10563353; doi:10.1186/s13561-023-00460-9)
Supplement: Supplementary file 1 — Supplementary Material 1 [file 13561_2023_460_MOESM1_ESM.pdf]

SUPPLEMENTARY MATERIALS

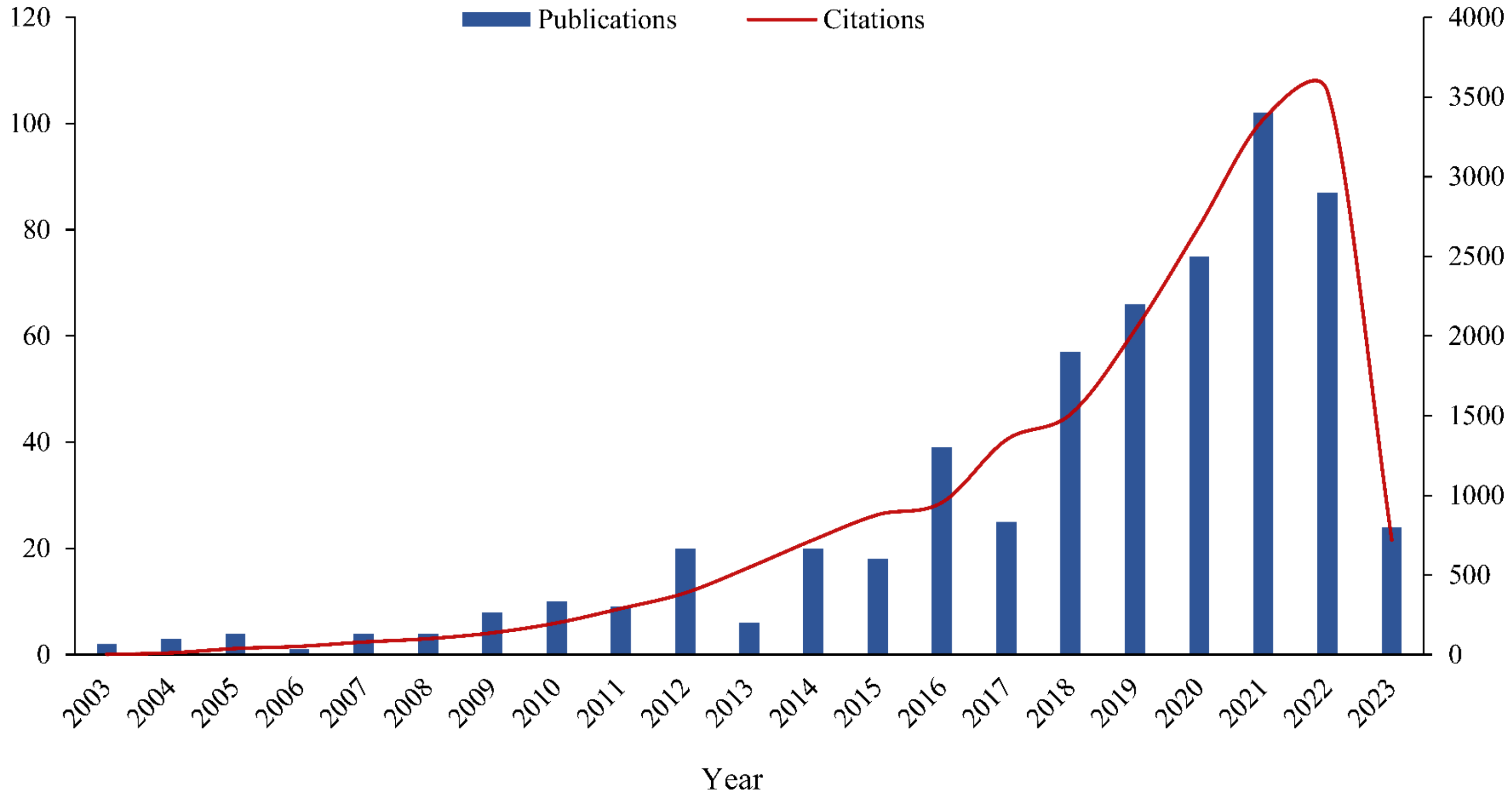

Figure S1. Number of publications and citations per year

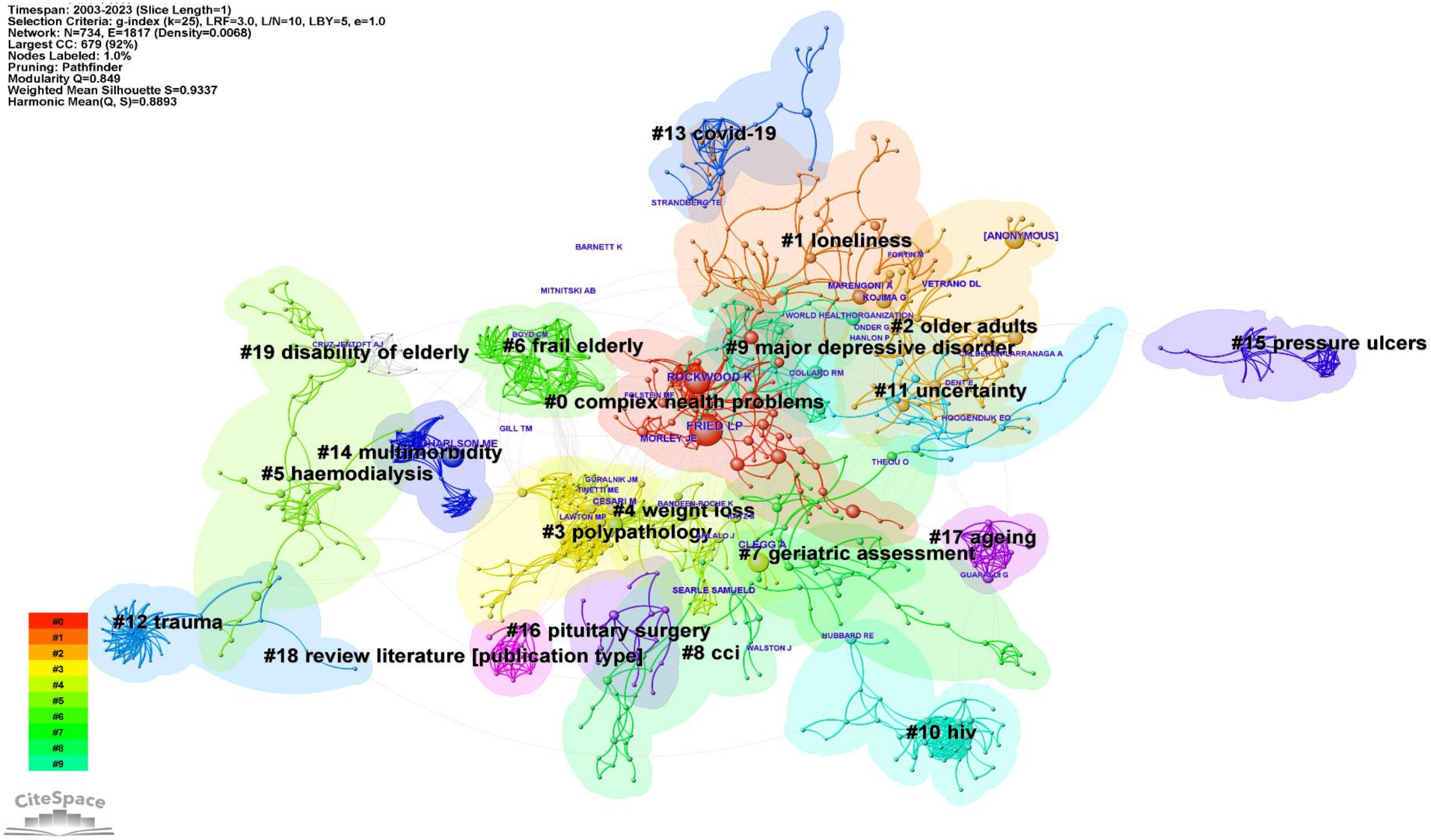

Figure S2. Co-cited authors network and corresponding clustering visualization generated by CiteSpace. Note: A node represents a cited author. The network is organized by the number of citations per node. The size of a node is proportional to its citations. Lines indicate co-citation relationships between authors. A large node indicates significant contributions and influence in the network.

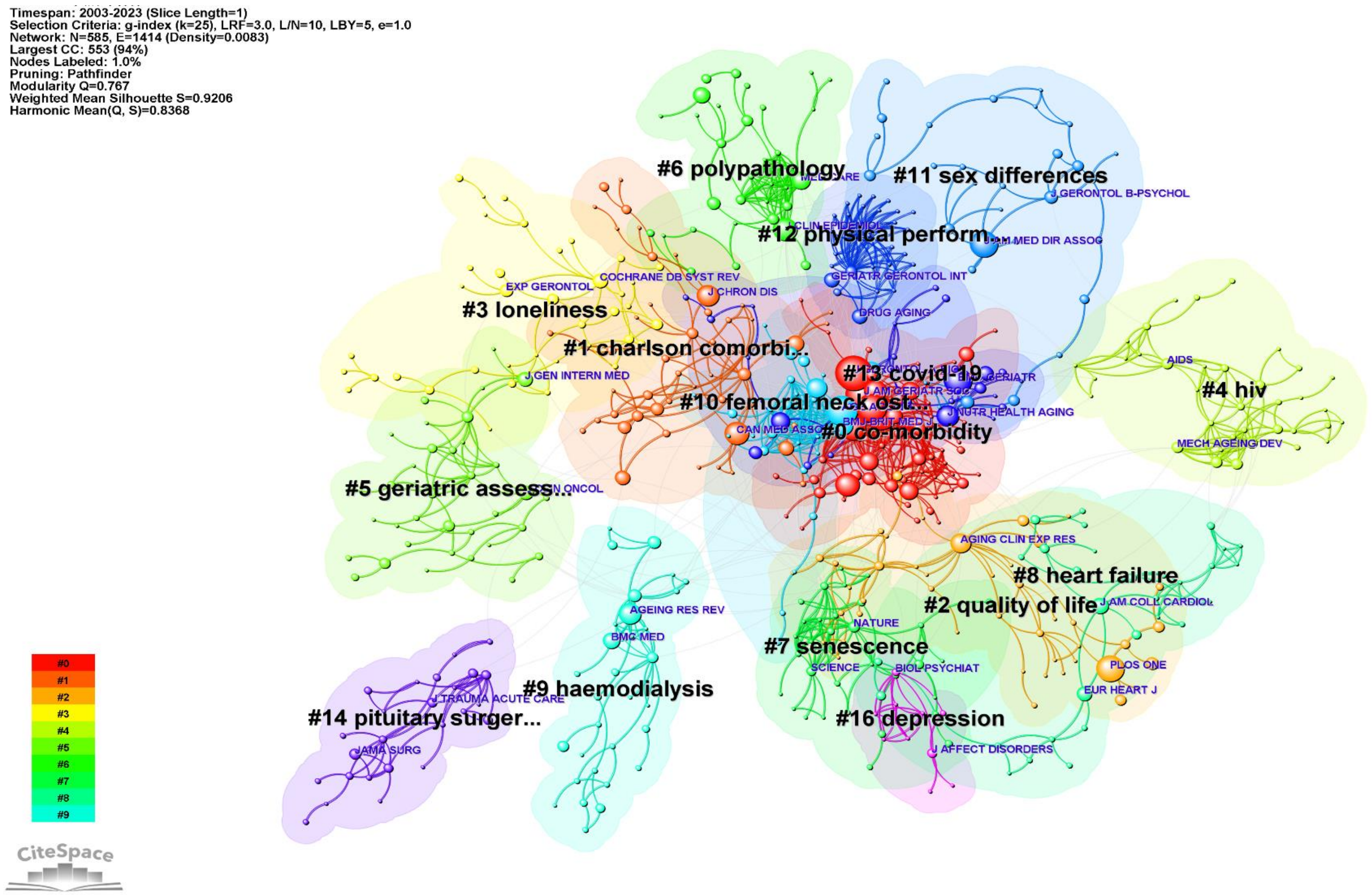

Figure S3. Co-cited journals network and corresponding clustering visualization generated by CiteSpace. Note: A node represents a cited journal. The network is organized by the number of citations per node. The size of a node is proportional to its citations. Lines indicate co-citation relationships between journals. A large node indicates significant contributions and influence in the network.

Table S1. Summary of the largest clusters identified for co-citation network of references

Cluster ID	Size	Silhouette	Mean (Year)	Top five extracted terms based on keywords (Log-likelihood ratio algorithm; *p*-level)
#0	81	0.93	2013	comprehensive geriatric assessment (10.99, 0.001); chronic obstructive pulmonary disease (7.31, 0.01); polypharmacy (4.96, 0.05); prevalence (4.95, 0.05); prevention (3.65, 0.1)
#1	79	0.854	2017	deprescribing (9.42, 0.005); haemodialysis (6.27, 0.05); kidney transplantation (6.27, 0.05); aging phenotype (6.27, 0.05); hand grip strength (6.27, 0.05)
#2	76	0.979	2007	co-morbidity (11.61, 0.001); disability (9.09, 0.005); multimorbidity (6.13, 0.05); influenza (6, 0.05); cmv (6, 0.05)
#3	70	0.851	2017	multimorbidity (14.4, 0.001); chronic diseases (6.29, 0.05); personalized medicine (6.29, 0.05); hiv (4.67, 0.05); chronic disease (3.65, 0.1)
#4	49	0.978	2015	modified frailty index (20.16, 1.0E-4); charlson comorbidity index (15.32, 1.0E-4); multimorbidity (10.24, 0.005); adverse outcomes (10.02, 0.005); elixhauser comorbidity measure (10.02, 0.005)
#5	48	0.93	2019	covid-19 (21.94, 1.0E-4); sars-cov-2 (17.64, 1.0E-4); geriatrics (9.35, 0.005); electronic frailty index (9.07, 0.005); risk assessment (4.52, 0.05)
#6	31	0.954	2013	HIV (10.8, 0.005); telomere (7.46, 0.01); toxicity (7.46, 0.01); senescence (7.46, 0.01); pharmacokinetics (7.46, 0.01)
#7	30	0.926	2018	depressive symptoms (5.62, 0.05); hiv (5.62, 0.05); transitions (5.62, 0.05); psychosocial (5.62, 0.05); neurocognitive impairment (5.62, 0.05)
#8	23	0.996	2010	morbidity trends (7.82, 0.01); complex health problems (7.82, 0.01); oldest old (7.82, 0.01); functional status assessment (7.82, 0.01); frail older adults (5.08, 0.05)
#10	16	0.994	2015	geriatric assessment (10.66, 0.005); geriatric syndrome (6.9, 0.01); old (6.9, 0.01); multidisciplinary care (6.9, 0.01); non-leukemic death (6.9, 0.01)
#13	13	0.987	2012	osteoporosis (10.29, 0.005); fractures (10.29, 0.005); aged (4.62, 0.05); comorbidities (3.53, 0.1); multimorbidity (0.77, 0.5)
#14	13	0.994	2006	frailty of elderly (10.8, 0.005); comorbidities in elderly (10.8, 0.005); marigliano-cacciafesta polypathogical scale (mcps) (10.8, 0.005); cumulative illness rating scale (cirs) (10.8, 0.005); frailty (1.24, 0.5)
#16	12	0.992	2007	comprehensive geriatric (8.41, 0.005); chemotherapy (8.41, 0.005); clinical trials (8.41, 0.005); assessment (8.41, 0.005); geriatric assessment (2.59, 0.5)
#24	7	0.991	2013	colombia (8.25, 0.005); readmission to hospital (8.25, 0.005); atrial fibrillation (8.25, 0.005); self-care (8.25, 0.005); bogota (8.25, 0.005)

Table S2. Top 22 references with the strongest citation bursts (Minimum 2-year burst duration)

References	Year	Strength	Begin	End	2003-2023
Vetrano DL, 2019, J GERONTOL A-BIOL, V74, P659, DOI 10.1093/gerona/gly110, DOI	2019	11.93	2020	2023	
Clegg A, 2013, LANCET, V381, P752, DOI 10.1016/S0140-6736(12)62167-9, DOI	2013	10.58	2014	2018	
Morley JE, 2013, J AM MED DIR ASSOC, V14, P392, DOI 10.1016/j.jamda.2013.03.022, DOI	2013	8.91	2015	2018	
Hoogendijk EO, 2019, LANCET, V394, P1365, DOI 10.1016/S0140-6736(19)31786-6, DOI	2019	7.48	2020	2023	
Hanlon P, 2018, LANCET PUBLIC HEALTH, V3, PE323, DOI 10.1016/S2468-2667(18)30091-4, DOI	2018	6.65	2020	2023	
Collard RM, 2012, J AM GERIATR SOC, V60, P1487, DOI 10.1111/j.1532-5415.2012.04054.x, DOI	2012	6.06	2015	2017	
Gilbert T, 2018, LANCET, V391, P1775, DOI 10.1016/S0140-6736(18)30668-8, DOI	2018	5.54	2021	2023	
Hewitt J, 2020, LANCET PUBLIC HEALTH, V5, PE444, DOI 10.1016/S2468-2667(20)30146-8, DOI	2020	5.38	2021	2023	
Bergman H, 2007, J GERONTOL A-BIOL, V62, P731, DOI 10.1093/gerona/62.7.731, DOI	2007	4.96	2007	2012	
Kojima G, 2018, AGE AGEING, V47, P193, DOI 10.1093/ageing/afx162, DOI	2018	4.94	2021	2023	
Bandeen-Roche K, 2015, J GERONTOL A-BIOL, V70, P1427, DOI 10.1093/gerona/glv133, DOI	2015	4.28	2019	2020	
Clegg A, 2016, AGE AGEING, V45, P353, DOI 10.1093/ageing/afw039, DOI	2016	4.07	2017	2021	
Subramaniam S, 2018, J AM COLL SURGEONS, V226, P173, DOI 10.1016/j.jamcollsurg.2017.11.005, DOI	2018	3.85	2020	2023	
Johnston MC, 2019, EUR J PUBLIC HEALTH, V29, P182, DOI 10.1093/eurpub/cky098, DOI	2019	3.69	2021	2023	
Soysal P, 2017, AGEING RES REV, V36, P78, DOI 10.1016/j.arr.2017.03.005, DOI	2017	3.53	2020	2023	
Cesari M, 2017, J AM MED DIR ASSOC, V18, P361, DOI 10.1016/j.jamda.2016.12.086, DOI	2017	3.52	2018	2020	
Barnett K, 2012, LANCET, V380, P37, DOI 10.1016/S0140-6736(12)60240-2, DOI	2012	3.51	2014	2017	
Nunes BP, 2016, ARCH GERONTOL GERIAT, V67, P130, DOI 10.1016/j.archger.2016.07.008, DOI	2016	3.39	2020	2021	
Walston J, 2006, J AM GERIATR SOC, V54, P991, DOI 10.1111/j.1532-5415.2006.00745.x, DOI	2006	3.28	2008	2009	
Palmer K, 2018, HEALTH POLICY, V122, P4, DOI 10.1016/j.healthpol.2017.09.006, DOI	2018	3.13	2018	2020	
Onder G, 2015, EUR J INTERN MED, V26, P157, DOI 10.1016/j.ejim.2015.02.020, DOI	2015	3.1	2018	2019	
Handforth C, 2015, ANN ONCOL, V26, P1091, DOI 10.1093/annonc/mdu540, DOI	2015	3.1	2018	2019	

Table S3. Top 10 citing articles with the strongest centrality divergence scores

Number of citations in our database	Centrality divergence	Author	Journal	Title	DOI
18	2.2481	Amici A, 2008	ARCH GERONTOL GERIAT	The Marigliano-Cacciafesta polypathological scale: A tool for assessing fragility	10.1016/j.archger.2007.05.007
70	2.0086	Sarkisian CA, 2008	J AM GERIATR SOC	Preliminary Evidence for Subdimensions of Geriatric Frailty: The MacArthur Study of Successful Aging	10.1111/j.1532-5415.2008.02041.x
32	0.5652	Wleklik M, 2020	FRONT PSYCHOL	Multidimensional Approach to Frailty	10.3389/fpsyg.2020.00564
52	0.0742	Ritt M, 2017	CLIN INTERV AGING	Two Years with COVID-19: The Electronic Frailty Index Identifies High-Risk Patients in the Stockholm Gero Covid Study	10.2147/CIA.S124342
0	0.0724	Mak JKL, 2022	GERONTOLOGY	Comparing the predictive accuracy of frailty, comorbidity, and disability for mortality: a 1-year follow-up in patients hospitalized in geriatric wards	10.1159/000527206
14	0.0644	Ritt M, 2016	Z GERONTOL GERIATR	Significance of frailty for predicting adverse clinical outcomes in different patient groups with specific medical conditions	10.1007/s00391-016-1128-8
0	0.0638	Sinclair AJ, 2022	J PERS MED	Multimorbidity, Frailty, and Diabetes in Older People-Identifying Interrelationships and Outcomes	10.3390/jpm12111911
6	0.0284	Strandberg TE, 2021	EUR GERIATR MED	Phenotypic frailty and multimorbidity are independent 18-year mortality risk indicators in older men The Helsinki Businessmen Study (HBS)	10.1007/s41999-021-00472-w
4	0.0241	Bekic S, 2021	HEALTHCARE-BASEL	Clusters of Physical Frailty and Cognitive Impairment and Their Associated Comorbidities in Older Primary Care Patients	10.3390/healthcare9070891
113	0.0147	Guaraldi G, 2015	AIDS	A frailty index predicts survival and incident multimorbidity independent of markers of HIV disease severity	10.1097/QAD.0000000000000753

The centrality divergence metric measures the structural variation caused by an article 'a' in terms of the divergence of the distribution of betweenness centrality of nodes in the baseline network. The centrality divergence metric is potentially valuable for detecting boundary-spanning activities at interdisciplinary levels.

Table S4. Summary of the largest clusters extracted for co-occurring author's keyword network

Cluster ID	Size	Silhouette	Mean (Year)	Top five extracted terms based on keywords (Log-likelihood ratio algorithm; *p*-level)
#0	46	0.9	2011	aging (12.43, 0.001); hiv (7.63, 0.01); deficit accumulation (7.34, 0.01); nursing homes (6.87, 0.01); index (6.18, 0.05)
#1	42	0.861	2014	frail elderly (20.87, 1.0E-4); multiple chronic conditions (13.31, 0.001); chronic diseases (11.73, 0.001); health care (9.29, 0.005); personalized medicine (7.63, 0.01)
#2	37	0.874	2014	multimorbidities (6.14, 0.05); frail and elderly patients (4.8, 0.05); proportional hazards models (4.8, 0.05); patient reported outcome measures (4.8, 0.05); biventricular pacing (4.8, 0.05)
#3	34	0.885	2009	cognitive impairment (14.29, 0.001); frailty of the elderly (10.97, 0.001); hip fractures (7.28, 0.01); shared decision-making (5.48, 0.05); percutaneous coronary intervention (5.48, 0.05)
#4	34	0.847	2018	palliative care (29.41, 1.0E-4); modified frailty index (14.9, 0.001); end of life (11.73, 0.001); charlson comorbidity index (11.39, 0.001); adverse outcomes (10.69, 0.005)
#5	33	0.89	2009	frailty of elderly (9.4, 0.005); disability (4.78, 0.05); modified frailty index (mfi) (4.7, 0.05); low-income countries (4.7, 0.05); vulvar cancer (4.7, 0.05)
#6	33	0.787	2018	pituitary surgery (13.86, 0.001); meta analysis (12.16, 0.001); activities of daily living (11.49, 0.001); critical care (8.44, 0.005); prospective studies (8.44, 0.005)
#7	32	0.84	2014	haemodialysis (9.51, 0.005); sarcopenia (8.53, 0.005); hand grip strength (6.75, 0.01); comprehensive geriatric assessment (cga) (6.62, 0.05); bioimpedance (6.62, 0.05)
#8	28	0.849	2016	atrial fibrillation (14.66, 0.001); risk stratification (14.16, 0.001); heart failure (8.29, 0.005); chronic inflammation (7.6, 0.01); low-dose computed tomography (7.07, 0.01)
#9	27	0.881	2014	critical care capacity (11.21, 0.001); multiple myeloma (7.52, 0.01); multimorbidity (6.89, 0.01); in-hospital mortality (5.92, 0.05); chemotherapy (5.92, 0.05)

Table S5. Burstness analysis of author keywords

A. Top 13 keywords with the strongest strength of citation bursts (Minimum 2-year burst duration)

Keywords	Year	Strength	Begin	End	2003-2023
elderly people	2003	**5.64**	2003	2017	
functional status	2003	**3.69**	2003	2014	
population	2005	**3.18**	2014	2016	
frail elderly	2003	**3.15**	2003	2013	
quality of life	2012	**2.84**	2012	2014	
women health	2006	**2.76**	2006	2016	
infection	2021	**2.76**	2021	2023	
ability	2015	**2.69**	2015	2018	
models	2019	**2.64**	2019	2020	
Charlson comorbidity index	2018	**2.62**	2021	2023	
frailty	2007	**2.57**	2012	2016	
united states	2014	**2.49**	2014	2018	
cohort	2008	**2.49**	2015	2016	

B. Top 13 keywords with the strongest beginning of citation busts (Minimum 2-year burst duration)

Keywords	Year	Strength	Begin	End	2003-2023
elderly people	2003	5.64	**2003**	2017	
functional status	2003	3.69	**2003**	2014	
frail elderly	2003	3.15	**2003**	2013	
women health	2006	2.76	**2006**	2016	
quality of life	2012	2.84	**2012**	2014	
frailty	2007	2.57	**2012**	2016	
population	2005	3.18	**2014**	2016	
united states	2014	2.49	**2014**	2018	
ability	2015	2.69	**2015**	2018	
cohort	2008	2.49	**2015**	2016	
models	2019	2.64	**2019**	2020	
infection	2021	2.76	**2021**	2023	
Charlson comorbidity index	2018	2.62	**2021**	2023	

Table S6. Top 10 countries and institutions, ranked by documents and citation counts from 2003-2023

Countries	Number of documents	Countries	Number of citations	Institutions	Number of documents	Institutions	Number of citations
USA	159	USA	9524	Johns Hopkins University	23	Johns Hopkins University	5264
Italy	96	Italy	5004	Karolinska Institute	23	University of Cattolica del Sacro Cuore	3327
England	69	Canada	4380	University of Cattolica del Sacro Cuore	21	Dalhousie University	2956
Netherlands	54	Germany	3472	Radboud University Nijmegen	16	NIA	2761
Spain	51	France	3284	Stockholm University	15	Wake Forest University	2684
Canada	46	Belgium	2787	Mcgill University	13	Columbia University	2637
Germany	46	Mexico	2365	University of Brescia	12	University of Toronto	2222
Sweden	38	Netherlands	2245	Kings Coll London	11	Karolinska Institute	793
France	37	Spain	1609	University of Groningen	11	Stockholm University	571
Australia	35	England	1508	University of Sydney	11	University of Brescia	524

Table S7. Top 10 cited authors and journals, ranked by citation counts and betweenness centrality from 2003-2023

Author	Number of citations in the network	Author	Betweenness centrality	Journal	Number of citations in the network	Journal	Betweenness centrality
Fried LP	280	Onder G	0.20	J AM GERIATR SOC	373	AGING CLIN EXP RES	0.33
Rockwood K	176	Inouye SK	0.18	J GERONTOL A-BIOL	371	ANN INTERN MED	0.29
Charlson ME	119	Mitnitski AB	0.17	LANCET	303	AGE AGEING	0.22
Clegg A	103	Lawton MP	0.17	AGE AGEING	278	AM J PUBLIC HEALTH	0.21
Anonymous	94	Charlson M	0.17	JAMA-J AM MED ASSOC	237	AM J MED	0.18
Morley JE	78	Palmer K	0.14	PLOS ONE	220	AM J CARDIOL	0.17
Kojima G	58	Ellis G	0.13	BMC GERIATR	215	AM J PHYS MED REHAB	0.16
Cesari M	55	Morley JE	0.12	J AM MED DIR ASSOC	206	BMJ-BRIT MED J	0.15
Vetrano DL	55	Cesari M	0.12	NEW ENGL J MED	193	ANN ONCOL	0.15
Searle Samueld	51	Boyd CM	0.12	J CLIN EPIDEMIOL	167	AM J PSYCHIAT	0.15

Table S8. Burstness analysis of cited authors

A. Top 15 cited authors with the strongest strength of citation bursts (Minimum 2-year burst duration)

Cited Authors	Year	Strength	Begin	End	2003-2023
Vetrano DL	2019	**8.83**	2020	2023	
Walston J	2008	**7.26**	2008	2016	
Katz S	2007	**7.12**	2007	2014	
Hoogendijk EO	2018	**6.87**	2020	2023	
Hanlon P	2020	**6.6**	2020	2023	
Folstein MF	2007	**6.1**	2007	2016	
Bergman H	2007	**5.8**	2007	2013	
Hewitt J	2019	**5.36**	2021	2023	
Kojima G	2017	**5.31**	2020	2023	
Hogan Davidb	2009	**4.68**	2009	2015	
Romero-Ortuno R	2017	**4.46**	2017	2019	
Lawton MP	2009	**4.4**	2009	2012	
Fortin M	2014	**4.36**	2014	2017	
Van Kanga	2010	**4.21**	2013	2019	
Boyd CM	2008	**4.09**	2008	2018	

B. Top 15 cited authors with the strongest beginning of citation bursts (Minimum 2-year burst duration)

Cited Authors	Year	Strength	Begin	End	2003-2023
Cohen HJ	2004	3.84	**2004**	2009	
Katz S	2007	7.12	**2007**	2014	
Folstein MF	2007	6.1	**2007**	2016	
Bergman H	2007	5.8	**2007**	2013	
Walston J	2008	7.26	**2008**	2016	
Boyd CM	2008	4.09	**2008**	2018	
Hogan Davidb	2009	4.68	**2009**	2015	
Lawton MP	2009	4.4	**2009**	2012	
Woods NF	2010	3.76	**2010**	2015	
Chang SS	2012	3.83	**2012**	2019	
Van Kanga	2010	4.21	**2013**	2019	
Fortin M	2014	4.36	**2014**	2017	
Santos-Eggimann B	2016	3.9	**2016**	2019	
Romero-Ortuno R	2017	4.46	**2017**	2019	
Vetrano DL	2019	8.83	**2020**	2023	

Table S9. Top 25 cited journals with the strongest citation bursts

A. Top 15 cited journals with the strongest strength of citation bursts (Minimum 3-year burst duration)

Cited Journals	Year	Strength	Begin	End	2003-2023
ARCH INTERN MED	2003	**13.89**	2004	2017	
GERONTOLOGIST	2003	**7.95**	2003	2012	
ANN INTERN MED	2004	**7.93**	2004	2015	
LANCET PUBLIC HEALTH	2020	**7.79**	2020	2023	
J PSYCHIAT RES	2007	**7.71**	2007	2016	
AM J MED	2004	**7.25**	2004	2011	
AM J PUBLIC HEALTH	2004	**5.62**	2004	2014	
NEW ENGL J MED	2003	**5.38**	2003	2015	
J GERONTOL B-PSYCHOL	2010	**5.35**	2010	2016	
SOC SCI MED	2010	**5.22**	2010	2014	
JAMA-J AM MED ASSOC	2003	**5.16**	2008	2013	
AM J CLIN NUTR	2005	**4.8**	2005	2011	
WORLD NEUROSURG	2020	**4.55**	2020	2023	
EUR J CANCER	2016	**4.36**	2016	2019	
AM J PHYS MED REHAB	2003	**4.26**	2003	2013	
BIOL PSYCHIAT	2003	**4.13**	2003	2016	
ANN EPIDEMIOL	2009	**4.04**	2009	2013	
ANN FAM MED	2010	**4.02**	2014	2016	
AGING-CLIN EXP RES	2003	**3.76**	2003	2011	
J ADV NURS	2012	**3.48**	2012	2016	
MD STATE MED J	2009	**3.36**	2009	2014	
PREV CHRONIC DIS	2016	**3.35**	2016	2019	
CIRCULATION	2007	**3.3**	2010	2012	
BRIT MED J	2003	**3.26**	2003	2012	
AGING CLIN EXP RES	2003	**3.21**	2007	2012	

B. Top 15 cited journals with the strongest strength of citation bursts (Minimum 3-year burst duration)

Cited Journals	Year	Strength	Begin	End	2003-2023
GERONTOLOGIST	2003	7.95	**2003**	2012	
NEW ENGL J MED	2003	5.38	**2003**	2015	
AM J PHYS MED REHAB	2003	4.26	**2003**	2013	
BIOL PSYCHIAT	2003	4.13	**2003**	2016	
AGING-CLIN EXP RES	2003	3.76	**2003**	2011	
BRIT MED J	2003	3.26	**2003**	2012	
ARCH INTERN MED	2003	13.89	**2004**	2017	
ANN INTERN MED	2004	7.93	**2004**	2015	
AM J MED	2004	7.25	**2004**	2011	
AM J PUBLIC HEALTH	2004	5.62	**2004**	2014	
AM J CLIN NUTR	2005	4.8	**2005**	2011	
J PSYCHIAT RES	2007	7.71	**2007**	2016	
AGING CLIN EXP RES	2003	3.21	**2007**	2012	
JAMA-J AM MED ASSOC	2003	5.16	**2008**	2013	
ANN EPIDEMIOL	2009	4.04	**2009**	2013	
MD STATE MED J	2009	3.36	**2009**	2014	
J GERONTOL B-PSYCHOL	2010	5.35	**2010**	2016	
SOC SCI MED	2010	5.22	**2010**	2014	
CIRCULATION	2007	3.3	**2010**	2012	
J ADV NURS	2012	3.48	**2012**	2016	
ANN FAM MED	2010	4.02	**2014**	2016	
EUR J CANCER	2016	4.36	**2016**	2019	
PREV CHRONIC DIS	2016	3.35	**2016**	2019	
LANCET PUBLIC HEALTH	2020	7.79	**2020**	2023	
WORLD NEUROSURG	2020	4.55	**2020**	2023	
